# The gap between calculated and actual calcium substitution during citrate anticoagulation in an immobilised patient on renal replacement therapy reflects the extent of bone loss – a case report

**DOI:** 10.1186/1471-2369-15-163

**Published:** 2014-10-04

**Authors:** Matthias Klingele, Sarah Seiler, Aaron Poppleton, Philip Lepper, Danilo Fliser, Roland Seidel

**Affiliations:** Department of Internal Medicine, Nephrology and Hypertension, Saarland University Medical Centre, Homburg-Saar, Germany; Department of Pneumology, Allergology, Artificial Ventilation and Environmental Medicine, Saarland University Medical Centre, Homburg-Saar, Germany; Department of Diagnostic and Interventional Radiology, Saarland University Medical Centre, Homburg-Saar, Germany

**Keywords:** Bone loss, Citrate anticoagulation, Immobilisation, Renal replacement therapy

## Abstract

**Background:**

Demineralisation and bone density loss during immobilisation are known phenomena. However information concerning the extent of calcium loss during immobilisation remains inconsistent within literature. This may explain why treatment of bone loss and prevention of further demineralisation is often initiated only when spontaneous bone fracture occurred.

Continuous renal replacement therapy is commonly utilised in critically ill patients with acute kidney injury requiring RRT. Regional anticoagulation with citrate for CRRT is well-established within the intensive care setting. Due to calcium free dialysate, calcium is eliminated directly as well as indirectly via citrate binding necessitating calcium substitution. In anuric patients declining calcium requirements over time reflect bone calcium liberation secondary to immobilisation. The difference between the expected and actual need for calcium infusion corresponds to calcium release from bone which is particularly impressive in patients exposed to long-term immobilisation and CRRT. We report a dialysis period in excess of 250 days with continuous renal replacement therapy and anticoagulation with citrate.

**Case presentation:**

We present a 30-year old male with prolonged multisystem organ failure after bilateral lung transplantation, in whom during a period of 254 days the cumulative difference between expected and actual need for calcium infusion was 14.25 mol, representing an estimated calcium loss of about 571 g. Comparison of computed tomographic imaging of the lower thoracic vertebrae over this period depicts a radiographically discernible decrease in bone density from 238 to 52 Hounsfield Units. The first spontaneous fracture occurred after 6 months of immobilisation. Despite subsequent treatment with bisphosphonates and androgen therapy resulting in an increase in bone density to 90 HU a further fracture occurred.

**Conclusion:**

In immobilised patients receiving CRRT and anticoagulation with citrate, decreasing need for calcium substitution reflects the degree of bone demineralisation corresponding with radiographic assessment of declining bone mineral density. Such a declining need for calcium substitution could be useful in clinical practice highlighting relevant bone loss which results in spontaneous fractures in immobilised critically ill patients.

## Background

Although demineralisation and bone loss secondary to long-term immobilisation in critically ill patients are known phenomena, in clinical practice bone loss is generally considered less important with respect to disease severity and the context of intensive care being responsible for immobilisation. Immobilisation results in bone loss [1] and may result in spontaneous fractures [2]. However, information about the degree of bone loss and the magnitude of corresponding calcium loss is somewhat inconsistent within literature, being quantified by two different approaches: 1) renal calcium loss calculated as the difference between increased urinary and faecal calcium excretion and net absorption [3, 4], with the assumption that this difference corresponds to bone demineralisation, 2) comparison of bone mineral density through x-ray absorptiometry (DXA) scans – the more commonly utilised method [4] - with a calculated total body calcium loss during immobilisation in healthy volunteers of ~0.7% per month [4].

Using CVVHD for continuous renal replacement therapy (multifiltrate CiCa, Fresenius, Bad Homburg) with regional citrate anticoagulation the concentration of ionised calcium in the extracorporeal circuit is reduced to 0.25-0.35 mmol/l through chelation with citrate. Calcium is effectively eliminated during dialysis. To restore a physiological calcium level (normal range 2.2-2.6 mmol/l), calcium has to be added prior to return of the dialysed blood to the patient’s circulation. In anuric patients with unchanged dietary calcium intake or intestinal loss, a declining need for calcium infusion to restore physiological serum calcium level corresponds to increasing bone calcium release secondary to immobilisation [2].

The current case analysis had two aims: 1) to assess calcium loss following immobilisation though declining calcium infusion requirements during CRRT with regional citrate anticoagulation, and 2) to verify this new method for estimation of calcium and bone loss during immobilisation by correlation with radiographically depicted bone demineralisation.

## Case presentation

A 30-year old male patient with cystic fibrosis underwent bilateral lung transplantation. Postoperative persistent pulmonary infection and multisystem organ failure ensued, with CVVHD initiated for acute renal failure. Graft failure of the left lung resulted in unilateral retransplantation. The patient remained immobile due to persistent infection and multiple organ dysfunctions. Spontaneous fracture of the left tibia occurred after 6 months of immobility. The following weeks saw a progressive respiratory and clinical deterioration resulting in the patient’s death.

### Clinical course and study results

**Figure 1:**
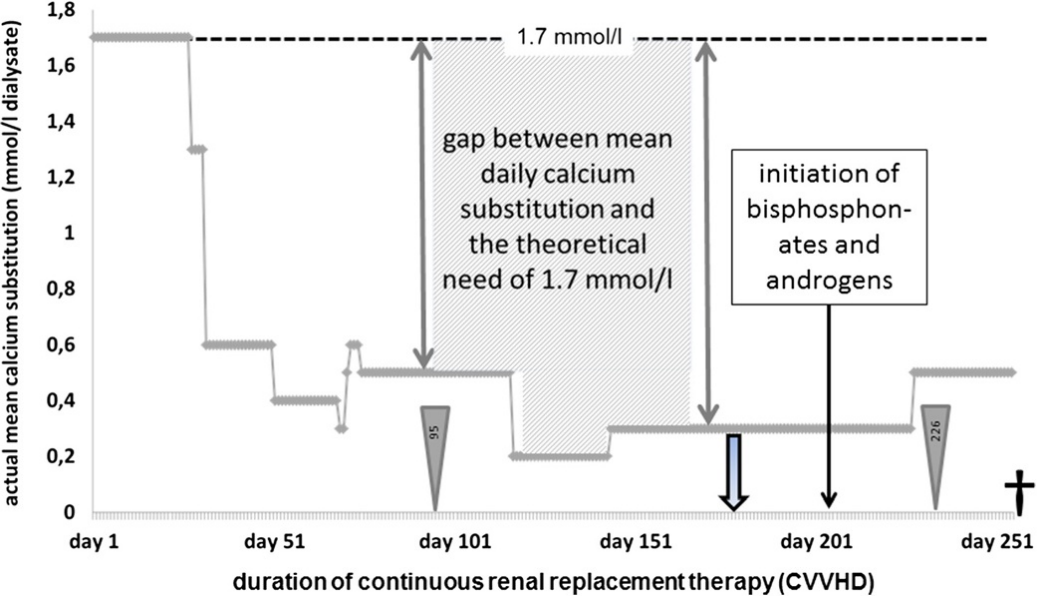
**Daily mean need for calcium substitution per litre dialysate.** Initial CVVHD required a calcium substitution of 1.7 mmol/l dialysate to maintain a serum ionised calcium level within physiological limits (dashed line). From day 27 of CVVHD requirement rapidly decreased. The lowest mean daily calcium substitution was 0.2 mmol calcium/l dialysate. The disparity between actual mean calcium substitution and the theoretical need of 1.7 mmol/l is shown for two days (grey flashes). The total disparity is the sum of these daily differences, shown in grey for a period of around 75 days. Broad arrows represent occurrence of spontaneous fractures. Computed tomographic imaging is indicated by grey triangles with related number showing days after start of dialysis. Death occurred at day 254 of CVVHD.

Total CVVHD duration was 254 days, with a mean dialysis period of 22.5 hours per day. Applied dialysis dose averaged at 37.8 ml/kg/h. For anticoagulation a mean of 4.0 mmol (3.4 to 4.3) citrate per litre blood was given, permitting a mean circuit lifespan of 64.4 hours. Mean calcium substitution was 0.5 mmol/l dialysate. Initial requirements of 1.7 mmol/l decreased after 27 days as shown in Figure 1. The lowest level of calcium substitution was 0.2 mmol/l. The calculated gap between actual calcium substitution and the theoretical need resulted in an estimated total calcium loss of 14.25 mol (about 571 g). Bisphosphonate therapy was initiated when spontaneous fracture occurred, and testosterone substituted after measurement of total testosterone level (0.76 ng/ml - normal range: 2.49-8.36 ng/ml).

At admission our patient showed normal renal function (eGFR (CKD-EPI) 143 ml/min, calculated GFR based on cystatin C 105 ml/min). Serum calcium measured within the normal range (2.5 mmol/l). Although iPTH was not determined at admission, pre-existing elevated iPTH levels had been noted. Parathyroid hormone levels were measured at day 152, 181, and 222 of CVVHD with values between 237–424 pg/ml (normal range: 10–50 pg/ml, Table 1). Serum calcitonin, vitamin A, and thyroid hormone levels remained within physiological parameters. Mean serum phosphate was 3.2 ± 0.84 mg/dl (1.03 ± 0.26 mmol/l). Initial vitamin D levels lay within the target range of >30 ng/ml and decreased over time despite substitution (Table 1).

**Figure 2:**
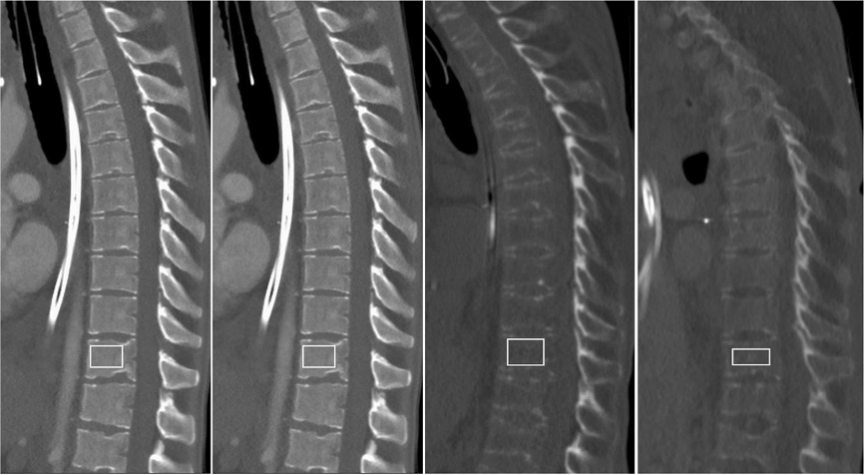
**Sagittal plain computed tomographic imaging of the vertebral column over time.** Computed tomographic imaging from four different time points are shown. Region of interest within the thoracolumbar vertebral body is marked with a white square. Corresponding Hounsfield Units (HU) at 3 years prior admission (242 HU), admission (238 HU), day 95 of CVVHD (52 HU), and day 226 of CVVHD (90 HU) are labelled accordingly. Three years prior to admission bone mineral density (BMD) was within physiological limits with age-adjusted configuration within normal limits for the thoracolumbar vertebrae. No significant change was found at admission. A significant decrease in BMD was noted at 4.5 months after admission (day 95 of CVVHD), with radiodensity decreasing to 52 HU preventing differentiation between the vertebral body edge and the spinal canal. BMD increased to 90 HU after intervention.

Computed tomographic imaging was performed at several points during the hospital stay. Bone density of the lower thoracic vertebrae was used as the basis for radiological comparison. Semi-quantitative assessment of bone demineralisation by radiodensity was described in Hounsfield Units (HU). Three years prior to admission thoracolumbar vertebrae showed a mineralisation with age-adjusted configuration within normal limits. At 242 HU bone mineral density (BMD) was within physiological limits (Figure 2). Although little change in BMD was seen at admission, a decrease to 52 HU was noted at 4.5 months after admission (day 95 of CVVHD). A parallel reduction in vertebral body height was clearly visible. Imaging 9 months after admission showed vertebral height remained unchanged, with an increase in BMD (90 HU) permitting differentiation between the trailing edge of the vertebral bodies and the spinal canal.

**Table 1:** **Serum levels of parathyroid hormone (PTH), vitamin D and ionized calcium**

		PTH						
**Day of CVVHD**	at admission (-43)	152	181	222				
**Serum level (pg/ml)**	not done	237	288	424				
**Ca ionised (mmol/l)**	1,18	1.13	1.05	1.07				
		**Vitamin D: 25(OH)D**						
**Day of CVVHD**	at admission (-43)	32	152	160	186	194	204	209
**Serum level (ng/ml)**	30.2	9.3	9.6	9.3	14.9	17.1	19.2	22.3
**Ca ionised (mmol/l)**	1,18	1.01	1.13	1.04	1.04	0.97	1.14	1.17

### Bone loss following immobilisation in literature

Published data concerning calcium loss secondary to bone demineralisation following immobilisation remain sparse and somewhat inconsistent within literature. Immobilisation trials amongst volunteers and astronauts have reported a calcium loss of 200-300 mg per day [5], and a renal urinary calcium excretion of up to 73 mmol per day (around 2.9 g per day) has been observed [6]. In studies measuring bone mineral content (BMC) and bone mineral density (BMD) by dual-energy X-ray absorptiometry [7–9] a decrease in bone density of between 0.5% and 1.3% per month of immobilisation is described [6]. However, no supportive data are available to allow estimation of real calcium loss with respect to radiological findings.

We therefore present an alternative means of estimating calcium loss in immobilised critically ill patients requiring dialysis. During CRRT and anticoagulation with citrate the gap between theoretical and actual calcium substitution represents the effective calcium loss [2], when intake and intestinal loss are similar and anuria prevents renal loss. In the current case a declining need for calcium supplementation occurred after 4 weeks, in keeping with publications reporting significant bone demineralisation after several weeks of immobilisation [10–13]. A cumulative estimated calcium loss of 571 g was noted over the 254 day period of CVVHD. This loss correlates with a semiquantative radiological measurement achieved through sequential comparison of bone density of the same skeletal region. As previous studies have reported total skeletal calcium content at 1–1.5 kg [14] the estimated calcium loss of 571 g reported here seems plausible, albeit with neither spontaneous fractures nor the semiquantative radiological assessment clinically proving this.

An average calcium loss of 2.2 g per day is however somewhat higher than observed in most previous studies. This could be accounted for by an accelerated bone loss secondary to immunosuppression used after lung transplantation i.e. calcineurin inhibitors and steroid therapy [15], the observed androgen deficiency [16], or the exceptionally long duration of immobilisation [7].

### Treatment of bone loss following immobilisation in literature

Bisphosphonates were given to stop bone calcium loss secondary to immobilisation, a practice first described in the 1970s [4]. With the intention of increasing bone mineralisation, androgens were administered as proposed by Gruenewald [16]. Together they resulted in a small increase in need for calcium substitution during CVVHD and anticoagulation with citrate, reflecting a reduction in bone calcium loss. A concomitant increase in bone radiodensity was noted.

### Long-term dialysis in literature

A total CVVHD duration of 254 days is exceptional compared to reported prolonged CRRT-durations of >20 days [2, 17]. As such, the extent to which citrate anticoagulation could promote bone demineralisation remains an important unanswered question. Heparin-based anticoagulation in CRRT is often contraindicated in the critically ill. Furthermore, an adequate dialysis dose effectively removes calcium meaning hypercalcaemia remains ‘invisible’ when heparin-based anticoagulation is used, masking a relative immobilisation hypercalcaemia [2]. Using citrate for anticoagulation a further negative calcium balance is maintained via continuous calcium losses in the CRRT effluent [2]. However, the declining need for substitution indicates indirectly the raising release of calcium from the bone – this would remain invisible using heparin for anticoagulation. Furthermore, long-term treatment with heparin can also result in osteoporosis [18].

### Limitations

Due to the study design, markers with respect to bone remodeling, bone formation, and bone resorption cannot be provided. Dietary calcium intake was assumed to be unchanged over time; however actual daily calcium intake was not recorded in detail.

## Conclusion

In immobilised patients receiving CRRT and regional citrate anticoagulation, decreasing need for calcium substitution may help estimate the degree of bone loss over time. Demineralisation and bone loss secondary to immobilisation in critically ill patients are generally considered less important with respect to disease severity and the context of intensive care. We feel that the suggested method would be helpful in bringing the problem of immobilisation associated bone loss to the foreground. Decreasing need for calcium substitution would act as warning sign, triggering investigation of potential physiological abnormalities e.g. androgen deficiency, and to consider initiation of bone protective measures to counter further bone loss. Such measures could prevent spontaneous fractures in long-term immobilised patients receiving CRRT and regional citrate anticoagulation. In light of this we recommend further evaluation of this proposed method.

## Consent

Written informed consent permitting publication of the above case was obtained from the legal guardian of the patient during the patient’s hospital stay.

## Abbreviations

BMD: Bone mineral density
CRRT: Continuous renal replacement therapy
CVVHD: Continuous venovenous haemodialysis
HU: Hounsfield units.
